# Decellularization of canine kidney for three-dimensional organ regeneration

**DOI:** 10.14202/vetworld.2020.452-457

**Published:** 2020-03-12

**Authors:** Kazuki Tajima, Kohei Kuroda, Yuya Otaka, Rie Kinoshita, Mizuki Kita, Toshifumi Oyamada, Kazutaka Kanai

**Affiliations:** 1 Department of Small Animal Internal Medicine II, School of Veterinary Medicine, Kitasato University, Towada, Japan; 2 Department of Surgery, Keio University School of Medicine, Shinjuku, Japan; 3 Department of Veterinary Pathology, School of Veterinary Medicine, Kitasato University, Towada, Japan

**Keywords:** bioengineering, dog, extracellular matrix, kidney, regeneration

## Abstract

**Background and Aim:**

Kidney regeneration is required for dogs with end-stage renal failure. Decellularization is one of the bioengineering techniques, which involves the removal of all tissue cells and cellular components and conservation of the extracellular matrix (ECM). Studies in rats have shown that decellularized kidney has regenerative potential; however, there are no reports on renal decellularization in dogs. Here, we showed the decellularization of the canine kidney.

**Materials and Methods:**

The renal artery of the cadaveric canine kidney was cannulated and the whole kidney was frozen at −80°C. After completely thawing, it was perfused with physiological saline and sodium dodecyl sulfate (0.5%, 6 h) through the cannulated renal artery to achieve decellularization. To assess the efficiency of the decellularization protocol, histological and immunohistochemical analysis of decellularized kidney was performed.

**Results:**

The results of hematoxylin and eosin (H and E) staining revealed that the decellularized canine kidney had no apparent cellular components. In addition, 4’,6-diamidino-2-phenylindole (DAPI) staining showed no visible nuclear components within the whole decellularized kidney. Therefore, both H and E and DAPI staining showed decellularization of the canine kidney. Our decellularization protocol also preserved the basement membrane of glomerulus, shown by periodic acid methenamine silver, periodic acid–Schiff, fibronectin, and collagen type IV stain.

**Conclusion:**

Our decellularization protocol could eliminate cellular components and remaining native ECM structures of canine kidney. These results could promote further research into canine kidney regeneration, which may be the first small step to regenerate the canine kidney waiting for renal transplantation.

## Introduction

Kidney disease is an important problem in dogs and cats. Chronic kidney disease (CKD) is the most common kidney disease in dogs and cats, and the estimated prevalence of CKD, according to one study in the UK, was 0.37% in dogs [1]. CKD is irreversible and progressive [2-5], which may eventually lead to a terminal loss of renal function. Apart from renal transplantation, there is no treatment to improve the renal function of patients with end-stage renal failure [6]. Some reports have indicated that old age and severe azotemia may increase mortality rates after renal transplantation [6,7]. Hence, renal transplantation in the early stages is desired to improve prognosis. However, the fundamental shortage of donors has limited renal transplantation in veterinary medicine. Therefore, organ regeneration could be possible therapeutics for animals with renal failure.

Decellularization is a unique procedure with a potential ability to overcome organ failure [8,9]. It involves the removal of all cells while preserving extracellular matrix (ECM) [10-12]. These organ-specific ECM proteins contribute to cell migration, proliferation, and differentiation [13-15]. Hence, these properties enable the decellularized scaffold of the organ to be a niche for the reseeded cells [8]. This bioscaffold also preserves the three-dimensional structure; therefore, organ regeneration could be achieved by recellularization of the original or immature cells [8].

Our group previously reported the decellularization of organs including the liver, heart, and pancreas in pigs, and these organs could achieve recellularization [15-18]. In addition, some reports indicated that the possibility of renal regeneration in rats using the decellularized kidney; this bioengineering technique also showed a potential for urine production in the recellularized kidney [14,19]. Furthermore, some groups have reported large-scale kidney decellularization in animals such as pigs and rhesus monkeys [14,20,21]. These reports showed that the recellularized organ can potentially regenerate the functional organ.

The protocol of whole-organ decellularization relies on the animal species and organ type. Various decellularization methods have been reported in the kidney [14,20,21]. Although freezing is widely used for physical decellularization, it could effectively disrupt the cellular membrane and induce cell lysis [11]. In addition, sodium dodecyl sulfate (SDS) has been reported as one of the most effective chemicals for decellularization, due to its ability to remove cellular components and remnants [11]. Hence, the combination of physical and chemical decellularization protocols is used for many organs [16-18]. However, decellularization of the canine kidney has not been reported.

In this study, we performed canine kidney decellularization using a cadaveric kidney. To the best of our knowledge, this is the first report on canine kidney decellularization.

## Materials and Methods

### Ethical approval

This experimental procedure to use canine cadaveric kidneys from euthanized dogs was approved by the Animal Care and Use Committee of Kitasato University (Approval No. 19-006).

### Harvest and storage of canine kidney

Kidneys of beagle dogs (n=3) euthanized for reasons not related to this study were obtained. The cadaveric kidneys were harvested within 12 h post-euthanasia. To harvest the cadaveric kidneys, all procedures were performed under sterile conditions. A midline incision was made to open the retroperitoneum. The renal artery, renal vein, and ureter were isolated. The renal artery was cannulated with an 18G indwelling needle and the renal vein and ureter were cut before harvesting the kidney from the canine’s body. The harvested kidneys (approximately 5-7 cm in the major axis) were either stored at −80°C until decellularization or were immediately used as control for histological analysis.

### Decellularization protocols

The decellularization method was slightly modified from the previous reports on porcine kidneys [20]. After completely thawing of the frozen canine kidney, physiological saline was perfused into the renal artery for 2 h using a peristaltic pump (Masterflex peristaltic tubing pumps; Cole-Parmer Instrument Company, USA) at a flow rate of 15 mL/min. Next, 0.5% SDS (Wako Pure Chemical Industries, Japan) was perfused for 6 h to decellularize the kidney. The decellularized kidney was subsequently perfused with saline for 2 h to remove the SDS solution and analyzed histologically. We confirmed the canine decellularization protocol in triplicate.

### Histological analysis

First, histological analysis of cadaveric and decellularized kidneys was performed. The obtained cadaveric and decellularized kidneys were immediately immersed and fixed in 4% paraformaldehyde in phosphate-buffered saline (PBS) for 24 h at 4°C. The fixed samples were embedded in paraffin and cut into 4 μm sections. Subsequently, they were deparaffinized in xylene, rehydrated in water, and stained with hematoxylin and eosin (H and E) or mounted with mounting medium 4’,6-diamidino-2-phenylindole (DAPI) (Vector Laboratories, Inc., USA). Using four random microscopic photographs of the cadaveric and decellularized kidney, the DAPI-positive area was measured by ImageJ software (National Institute of Health, Bethesda, USA). In addition, the decellularized kidney sections also stained with periodic acid methenamine silver (PAM) and periodic acid–Schiff (PAS) by standard protocol [22] and observed by microscopy.

### Immunohistochemical analysis

Paraffin-embedded sections of decellularized kidney were also used for immunohistochemical analysis including fibronectin (Merck K.K., Tokyo, Japan) and collagen type IV (LifeSpan Biosciences, Inc., Washington, USA). In brief, the slides were rehydrated in the same way as histological analysis. To perform diaminobenzidine staining, the tissue was incubated with H_2_O_2_ for 30 min for removing endogenous staining. Antigen retrieval was performed by microwave for 20 min, and non-specific binding of antibodies was blocked by normal goat serum (Agilent Technologies Japan, Ltd., Tokyo, Japan). Then, the slides were incubated with anti-fibronectin antibody (1:100) for overnight at 4°C. After washing with PBS, the EnVision labeled polymer-horseradish peroxidase system (Agilent Technologies Japan, Ltd.) was used as the secondary antibody. Peroxidase activity was detected with diaminobenzidine substrate (Agilent Technologies Japan, Ltd.). Alternatively, collagen type IV was detected by immunofluorescence staining. Sections were treated for heat-mediated antigen retrieval in citrate buffer at pH 6.0, and non-specific reaction of primary antibody was blocked by goat serum. Primary antibody for collagen type IV was diluted 50:1 and incubated for 1 h at room temperature. After rinsing, collagen type IV-stained sample was incubated with secondary antibody labeled with Alexa 488 for 1 h at room temperature. These samples were mounted and observed using light and fluorescence microscopy.

### Statistical analysis

Data were expressed as mean±standard devition (SD). To compare the nuclear staining positive areas between cadaveric and decellularized kidneys, Mann–Whitney non-parametric test was performed by GraphPad Prism software (version 7.0d for Mac, La Jolla, USA). p<0.05 was considered to be statistically significant.

## Results

**Figure-1:**
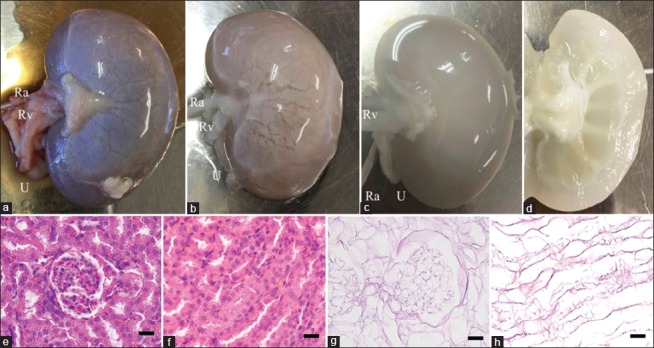
Representative gross and histological photograph of the canine kidney during decellularization. The cadaveric canine kidney after thawing and before perfusion (a). Canine kidney during sodium dodecyl sulfate perfusion through renal artery (b). Canine decellularized kidney immediately after complete perfusion of all solutions (c). Ra=Renal artery, Rv=Renal vein. U=Ureter. Cross-section of canine kidney after decellularization (d). H and E staining of cadaveric (e and f) and decellularized (g and h) canine kidney. H and E staining shows glomerular (e and g) and tubular (f and h) structure. All images, scale bars: 25 μm. H and E=Hematoxylin and eosin.

To prove the efficiency of our decellularization protocol, the cadaveric canine kidney was evaluated by macrophotography during decellularization. Immediately after thawing the cadaveric kidney, the kidney contained blood and appeared reddish-brown (Figure-1a). Physiological saline was then perfused through the renal artery to wash out the blood within the cadaveric kidney. Finally, to remove all renal cells, SDS solution was perfused for 6 h. Subsequently, the kidney color gradually became brighter during decellularization (Figure-1b). In addition, after SDS perfusion, the whole kidney changed from white to translucent (Figure-1c and d). Through macrophotographic assessment, our SDS-based protocol might achieve decellularization of the canine kidney, while maintaining three-dimensional kidney structures.

Besides the gross changes of the kidney after decellularization, we also assessed the effectiveness of our decellularization protocol by H and E staining. The cadaveric kidney showed dense renal cells, including the glomerulus and tubules (Figure-1e and f). Conversely, the decellularized kidney showed no apparent cellular components within the whole canine kidney; however, renal ECM, including the basement membrane of the glomerulus, Bowman’s capsule, and tubular structures, was present in the decellularized kidney (Figure-1g and h). These results indicated that our decellularization protocol removed almost cellular components within the canine kidney at the microscopic level, while preserving renal microstructures.

**Figure-2:**
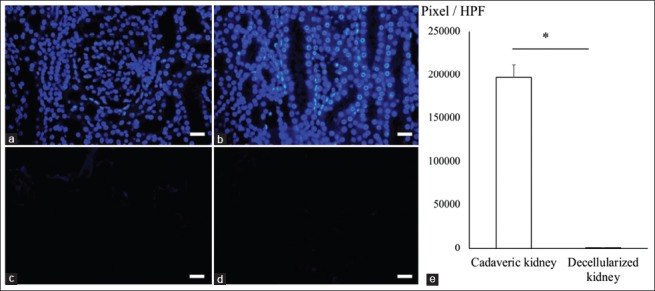
Analysis of DAPI staining in the decellularized canine kidney. DAPI staining of cadaveric (a and b) and decellularized (c and d) canine kidney. DAPI staining shows glomerular (a and c) and tubular (b and d) structure. DAPI-positive area was compared between the cadaveric and decellularized kidney (e). The graph shows mean±standard deviation. All images, scale bars: 25 μm. *p<0.05. HPF=High-power focus, DAPI=4’,6-diamidino-2-phenylindole.

To assess the removal of nuclear remnants within our decellularized canine kidney, DAPI staining of the cadaveric and decellularized kidney was performed. The result showed that the cadaveric kidney maintained ubiquitous existence of DAPI-positive renal cells (Figure-2a and b). Conversely, the decellularized kidney showed no visible DAPI staining in the sectioned area of the kidney (Figure-2c and d). The DAPI-positive area between cadaveric and decellularized kidneys was compared using randomized high-power focus (HPF) images (Figure-2e). This comparison revealed that the decellularized kidney had a significantly reduced DAPI-positive area (460.75±374.89 pixel/HPF; mean±SD) compared to the cadaveric kidney (196844.25±15107.85 pixel/HPF, p=0.0286, Mann–Whitney U-test). These results indicated that our decellularization protocol removed most DNA contents within the decellularized canine kidney.

**Figure-3:**
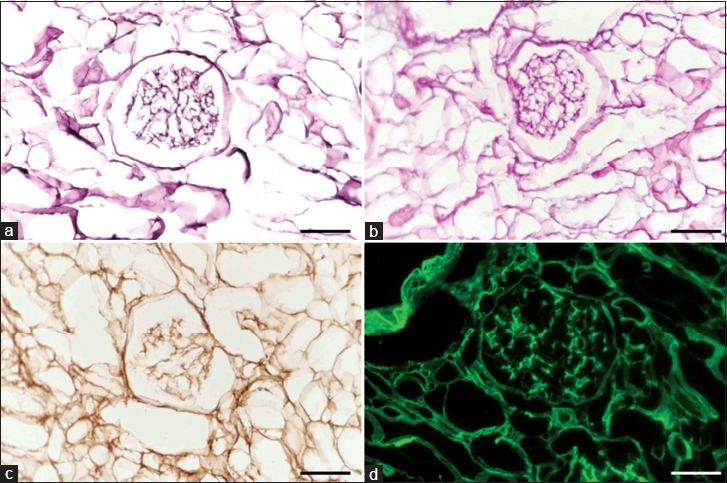
Staining of extracellular matrix in the decellularized canine kidney. Periodic acid methenamine silver staining of decellularized canine kidney (a). Periodic acid–Schiff staining of decellularized canine kidney (b). Fibronectin (c) and collagen type IV (d) staining of decellularized kidney by immunohistochemical analysis. All images, scale bars: 50 μm.

Finally, we tried to detect ECMs within the decellularized canine kidney. PAM staining of decellularized kidney showed maintenance of basement membrane or reticulum fibers around the glomerular structure (Figure-3a). PAS staining of decellularized kidney showed remaining of mucopolysaccharides (Figure-3b), which is contained in the basement membrane. Furthermore, decellularized kidney demonstrated positive expression of fibronectin (Figure-3c) and collagen type IV (Figure-3d) within the basement membrane by immunohistochemical analysis. Above all, the decellularized kidney demonstrated that remaining of basic ECMs including fibronectin and collagen type IV.

## Discussion

Decellularization is one of the unique bioengineering techniques with the potential to regenerate three-dimensional organs [9]. In this study, we decellularized the canine kidney using a commonly used protocol, which involved a combination of the freezing and SDS perfusion methods.

Our decellularization protocol achieved gross architectural changes in the cadaveric canine kidney. This white to translucent color change provides an indication of decellularization quality. Histological analysis also supported this gross change of the kidney. H and E and DAPI staining showed no visible cellular and nuclear components within the decellularized scaffold except for organ-derived ECM; this indicates the robustness of our decellularization method. In addition to the removal of the cellular components, our protocol also preserved the renal microstructures such as glomerulus and tubules basement membrane. Above all, our decellularization protocol achieved the elimination of all cellular components and the remaining native ECM structures.

In this study, we freeze the cadaveric kidney as a decellularized protocol. Furthermore, we successfully decellularized the kidney from cadaveric dogs. This protocol has an advantage for storage the cadaveric kidney until decellularization. The obtaining donation of canine kidney could be preserved and used for decellularization by freezing. However, detailed verification of available harvested and preserved period is needed.

Complete decellularization is confirmed with (1) lack of nuclear materials in histologic analysis with H and E and DAPI staining, (2) residual DNA content should not exceed 200 base pair fragment length, and (3) the amount of dsDNA should not exceed 50 ng/mg of dry weight [23]. This study did not measure DNA level within the canine decellularized kidney. Before applying clinical transplantations, DNA concentration in the decellularized kidney should be clarified and further research is needed. Alternatively, in this study, we observed histological decellularization. This finding could allow for the achievement of low immunogenicity and facilitate renal transplantation without immunosuppressive drugs when the bioscaffold reseeded with low immunogenic cells such as induced pluripotent stem cells derived from renal cells. In future, the expansion culture of definitive cell source needs scaffold to accomplish three-dimensional organ regeneration. Our decellularized bioscaffold would be suitable for these cells.

In addition, decellularized bioscaffold provides appropriate niche for cell differentiation. Therefore, some established stem cells such as mesenchymal stem cells were known to have the potential to differentiate into functional cells within the decellularized scaffold [24,25]. ECMs are known to be important components of niche [26]. These reports indicated that our canine decellularized kidney, which remaining important basement membrane such as fibronectin and collagen type IV, might have the potential to differentiate into the functional renal cells using these stem cells. Further experiments are needed.

## Conclusion

We have achieved the decellularization of the canine kidney with preserving renal microstructures and ECMs. This result of basic research could promote further approach into kidney regeneration; this may be the first step to regenerate the canine kidney waiting for renal transplantation.

## Authors’ Contributions

KT and KKu designed the original idea for the study and drafted the manuscript. KKu and RK harvested the samples and performed the experiments. YO and MK performed histological evaluation and data analysis. TO and KKa supervised the study and reviewed the manuscript. KT performed the statistical analysis. All authors read and approved the final manuscript.

## References

[1] O'Neill D.G, Elliott J, Church D.B, McGreevy P.D, Thomson P.C, Brodbelt D.C (2013). Chronic kidney disease in dogs in UK veterinary practices:Prevalence, risk factors, and survival. J. Vet. Intern. Med.

[2] Polzin D.J (2011). Chronic kidney disease in small animals. Vet. Clin. North Am. Small Anim. Pract.

[3] Bartges J.W (2012). Chronic kidney disease in dogs and cats. Vet. Clin. North Am. Small Anim. Pract.

[4] Churchill J, Polzin D, Osborne C, Adams L (1992). The influence of dietary protein intake on progression of chronic renal failure in dogs. Semin. Vet. Med. Surg(Small Anim).

[5] Syme H.M, Markwell P.J, Pfeiffer D, Elliott J (2006). Survival of cats with naturally occurring chronic renal failure is related to severity of proteinuria. J. Vet. Intern. Med.

[6] Schmiedt C.W, Holzman G, Schwarz T, McAnulty J.F (2008). Survival, complications, and analysis of risk factors after renal transplantation in cats. Vet. Surg.

[7] Hopper K, Mehl M.L, Kass P.H, Kyles A, Gregory C.R (2012). Outcome after renal transplantation in 26 dogs. Vet. Surg.

[8] Song J.J, Ott H.C (2011). Organ engineering based on decellularized matrix scaffolds. Trends Mol. Med.

[9] Shaheen M.F, Joo D.J, Ross J.J, Anderson B.D, Chen H.S, Huebert R.C, Li Y, Amiot B, Young A, Zlochiver V, Nelson E, Mounajjed T, Dietz A.B, Michalak G, Steiner B.G, Davidow D.S, Paradise C.R, van Wijnen A.J, Shah V.H, Liu M, Nyberg S.L (2019). Sustained perfusion of revascularized bioengineered livers heterotopically transplanted into immunosuppressed pigs. Nat Biomed Eng.

[10] Crapo P.M, Gilbert T.W, Badylak S.F (2011). An overview of tissue and whole organ decellularization processes. Biomaterials.

[11] Gilbert T.W, Sellaro T.L, Badylak S.F (2006). Decellularization of tissues and organs. Biomaterials.

[12] Damodaran R.G, Vermette P (2018). Tissue and organ decellularization in regenerative medicine. Biotechnol. Prog.

[13] Bissell M.J, Aggeler J (1987). Dynamic reciprocity:How do extracellular matrix and hormones direct gene expression?. Prog Clin. Biol. Res.

[14] Song J.J, Guyette J.P, Gilpin S.E, Gonzalez G, Vacanti J.P, Ott H.C (2013). Regeneration and experimental orthotopic transplantation of a bioengineered kidney. Nat. Med.

[15] Yagi H, Fukumitsu K, Fukuda K, Kitago M, Shinoda M, Obara H, Itano O, Kawachi S, Tanabe M, Coudriet G.M, Piganelli J.D, Gilbert T.W, Soto-Gutierrez A, Kitagawa Y (2013). Human-scale whole-organ bioengineering for liver transplantation:A regenerative medicine approach. Cell Transplant.

[16] Katsuki Y, Yagi H, Okitsu T, Kitago M, Tajima K, Kadota Y, Hibi T, Abe Y, Shinoda M, Itano O, Takeuchi S, Kitagawa Y (2016). Endocrine pancreas engineered using porcine islets and partial pancreatic scaffolds. Pancreatology.

[17] Kitahara H, Yagi H, Tajima K, Okamoto K, Yoshitake A, Aeba R, Kudo M, Kashima I, Kawaguchi S, Hirano A, Kasai M, Akamatsu Y, Oka H, Kitagawa Y, Shimizu H (2016). Heterotopic transplantation of a decellularized and recellularized whole porcine heart. Interact. Cardiovasc. Thorac. Surg.

[18] Tajima K, Yagi H, Kitagawa Y (2018). Human-scale liver harvest and decellularization for preclinical research. Methods Mol. Biol.

[19] Du C, Narayanan K, Leong M.F, Ibrahim M.S, Chua Y.P, Khoo V.M, Wan A.C (2016). Functional kidney bioengineering with pluripotent stem-cell-derived renal progenitor cells and decellularized kidney scaffolds. Adv. Healthc. Mater.

[20] Poornejad N, Frost T.S, Scott D.R, Elton B.B, Reynolds P.R, Roeder B.L, Cook A.D (2015). Freezing/thawing without cryoprotectant damages native but not decellularized porcine renal tissue. Organogenesis.

[21] Nakayama K.H, Batchelder C.A, Lee C.I, Tarantal A.F (2010). Decellularized rhesus monkey kidney as a three-dimensional scaffold for renal tissue engineering. Tissue Eng. Part A.

[22] Prophet E.B (1992). and Armed Forces Institute of Pathology US. Laboratory Methods in Histotechnology.

[23] Londono R, Badylak S.F (2015). Biologic scaffolds for regenerative medicine:Mechanisms of *in vivo* remodeling. Ann. Biomed. Eng.

[24] Shimomura K, Rothrauff B.B, Tuan R.S (2017). Region-specific effect of the decellularized meniscus extracellular matrix on mesenchymal stem cell-based meniscus tissue engineering. Am. J. Sports Med.

[25] Li Y, Wu Q, Wang Y, Li L, Chen F, Shi Y, Bao J, Bu H (2017). Construction of bioengineered hepatic tissue derived from human umbilical cord mesenchymal stem cells via aggregation culture in porcine decellularized liver scaffolds. Xenotransplantation.

[26] Lukjanenko L, Jung M.J, Hegde N, Perruisseau-Carrier C, Migliavacca E, Rozo M, Karaz S, Jacot G, Schmidt M, Li L, Metairon S, Raymond F, Lee U, Sizzano F, Wilson D.H, Dumont N.A, Palini A, Fassler R, Steiner P, Descombes P, Rudnicki M.A, Fan C.M, von Maltzahn J, Feige J.N, Bentzinger C.F (2016). Loss of fibronectin from the aged stem cell niche affects the regenerative capacity of skeletal muscle in mice. Nat. Med.

